# NF-κB signaling mediates acquired resistance after PARP inhibition

**DOI:** 10.18632/oncotarget.2868

**Published:** 2015-01-13

**Authors:** Yuko Nakagawa, Anna S. Sedukhina, Naoki Okamoto, Satoi Nagasawa, Nao Suzuki, Tomohiko Ohta, Hiroyoshi Hattori, Marta Roche-Molina, Ana J. Narváez, Anand D. Jeyasekharan, Juan A. Bernal, Ko Sato

**Affiliations:** ^1^ Department of Translational Oncology, St. Marianna University Graduate School of Medicine, Kawasaki 216–8511, Japan; ^2^ Department of Obstetrics and Gynecology, St. Marianna University Graduate School of Medicine, Kawasaki 216–8511, Japan; ^3^ Division of Breast and Endocrine Surgery, Department of Surgery, St. Marianna University Graduate School of Medicine, Kawasaki 216–8511, Japan; ^4^ Laboratory of Advanced Therapy, Department of Hematology and Oncology Research, Clinical Research Center, National Hospital Organization Nagoya Medical Center, Nagoya 460–0001, Japan; ^5^ Department of Cardiovascular Development and Repair, Centro Nacional de Investigaciones Cardiovasculares (CNIC), Madrid 28029, Spain; ^6^ MRC Cancer Unit at the University of Cambridge, Hutchison Research Centre, CB2 0XZ, UK; ^7^ Cancer Science Institute of Singapore, National University of Singapore, Centre for Translational Medicine, 117599, Singapore

## Abstract

PARP inhibitors are a class of promising anti-cancer drugs, with proven activity in *BRCA* mutant cancers. However, as with other targeted agents, treatment with PARP inhibitors generates acquired resistance within these tumors. The mechanism of this acquired resistance is poorly understood. We established cell lines that are resistant to PARP inhibitor by continuous treatment with the drug, and then used RNA sequencing to compare gene expression. Pathway analysis on the RNA sequencing data indicates that NF-κB signaling is preferentially up-regulated in PARP inhibitor-resistant cells, and that knockdown of core components in NF-κB signaling reverses the sensitivity to PARP inhibitor in resistant cells. Of therapeutic relevance, we show that PARP inhibitor-resistant cells are sensitive to an NF-κB inhibitor in comparison to their parental controls. Malignancies with up-regulation of NF-κB are sensitive to bortezomib, a proteasome inhibitor that is currently used in the clinic. We also show that treatment with bortezomib results in cell death in the PARP inhibitor-resistant cells, but not in parental cells. Therefore we propose that up-regulation of NF-κB signaling is a key mechanism underlying acquired resistance to PARP inhibition, and that NF-κB inhibition, or bortezomib are potentially effective anti-cancer agents after the acquisition of resistance to PARP inhibitors.

## INTRODUCTION

Patients with the hereditary breast and ovarian cancer syndrome (HBOCS) commonly have mutations in the key genome stability proteins, *BRCA1* and *BRCA2*. Research over the last decade has yielded a promising therapeutic strategy for *BRCA* mutant cancers, through the observation that cells mutant for the *BRCA* genes are exquisitely sensitive to inhibition of the nuclear enzyme poly-adenosine ribose polymerase (PARP), through a synthetic lethal mechanism. These observations have been borne out in early phase clinical trials, with promising activity of PARP inhibitors both in breast and ovarian cancers [1–3]. In ovarian cancer, a recent phase II study has demonstrated a benefit of maintenance PARP inhibition in the management of metastatic ovarian cancers [4]. As with all maintenance therapeutic strategies, the development of resistance to prolonged single agent therapy is inevitable, thus necessitating the study of mechanisms of resistance and the development of therapeutic strategies to overcome them. Currently explored mechanisms for acquired resistance to PARP inhibition include 1. Reversion of the mutation of *BRCA* gene [5, 6], 2. Disruption of 53BP1 [7], 3. Up-regulation of p-glycoprotein efflux pump [8] and 4. Phosphorylation of ribosomal protein S6 [9]. However, there are no reports to date of a comprehensive screening approach to investigate the mechanism of resistance to PARP inhibition, especially in the context of ovarian cancer where maintenance PARP inhibitor therapy is of clinical benefit.

In this paper, we describe our studies comparing PARP inhibitor resistant and sensitive clones, and show an up-regulation of Nuclear Factor- κB (NF-κB) pathways in the resistant clones. NF-κB is a complex of transcription factors that consisting of p65 (RelA) and p50 (NFκB1) or RelB and p52 (NFκB2), that are known to function in the development of acquired resistance to several other targeted agents [10]. NF-κB signaling has two major pathways, one is the canonical pathway that mainly modulates cell proliferation, inflammation or anti-apoptosis, and the other one is the non-canonical pathway that mainly controls lymphogenesis and B cell maturation [11]. In the canonical pathway, p65/p50 NF-κB complex are localized in cytoplasm with IκB. Stimuli such as infection, cytokines, apoptosis-inducers activate NF-κB in canonical pathway. Binding those stimuli to their receptors including tumor necrosis factor receptor (TNFR) or interleukin 1 (IL-1) receptor (IL-1R) activates the IκB kinase (IKK) complex. The activated IκB kinase complex phosphorylates IκB and the phosphorylated IκB is degraded by β-TRCP-dependent ubiquitination. This results in nuclear translocation of p65/p50 heterodimer and activates transcription of NF-κB target genes [10]. In non-canonical pathway, p100, a precursor of p52, is a central player. p100 binds to RelB and stays in cytoplasm in non-activated state. Once activated via a binding of ligands including BAFF (B cell activating factor, a family member of TNF) to their receptors, p100 is processed to p52 and RelB/p52 heterodimer is translocated into nucleus to activate transcription of NF-κB target genes [12].

NF-κB inhibition rescues the sensitivity to anti-cancer drug in chemoresistant cancer cells, through TNFα mediated apoptosis, and indeed increases tumor regression [13]. Thus, NF-κB plays an important role in chemoresistance, and our paper describes a new role for this pathway in mediating resistance to PARP inhibition as well.

## RESULTS

### Establishment of PARP inhibitor resistant clone

**Figure 1:**
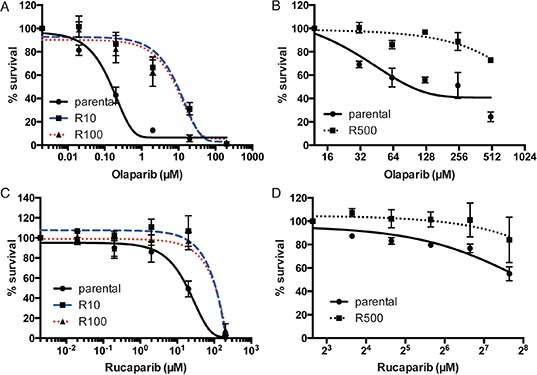
Sensitivity to PARP inhibitor in UWB1.289 cells Line chart shows the sensitivity to olaparib **(A)** and **(B)** or rucaparib **(C)** and **(D)** in parental or PARP inhibitor-resistant UWB1.289 cells **(A)** and **(C)** and in HCC1937 cells **(B)** and **(D)**. Error bar shows standard deviation of three independent experiments.

We used UWB1.289 ovarian cancer cells and HCC1937 breast cancer cells as parental cell lines to generate PARP inhibitor-resistant lines. Both the cell lines harbor homozygous mutation of *BRCA1*. PARP inhibitor-resistant clones (R10 and R100 in UWB1.289 and R500 in HCC1937) were developed independently by repeated exposure to different doses of PARP inhibitor (Olaparib AZD2281, KU-0059436) (10nM, 100nM and 500nM, respectively). To generate PARP inhibitor-resistant lines, we have used olaparib (AstraZeneca), a PARP inhibitor that is most advanced in clinical development, and currently in phase III testing [1–4]. For checking resistance to PARP inhibition, we used 2 distinct compounds-olaparib and rucaparib (AG014699); Clovis). While olaparib showed promising results in phase II studies in patients with breast and ovarian cancers having *BRCA* mutations [4], rucaparib was initially established as a radiosensitizer and to potentiate the effect of temozolamide. Phase II/III trials are currently underway for both these agents with preliminary results of activity in BRCA mutant cancers [14]. Both the olaparib and rucaparib are bona fide PARP inhibitors and inhibit both PARP1 and PARP2 [14]. Both of the compounds have similar potency in inhibiting PARP catalytic activity [14–16]. The PARP inhibitor-resistant lines show decreased sensitivity to both olaparib and rucaparib, both in UWB1.289 setting-R10/R100 (Figure 1A and 1B) and in the HCC1937 setting-R500 (Figure 1C and 1D).

### Validation of reported mechanisms for PARP inhibitor-resistance

**Figure 2:**
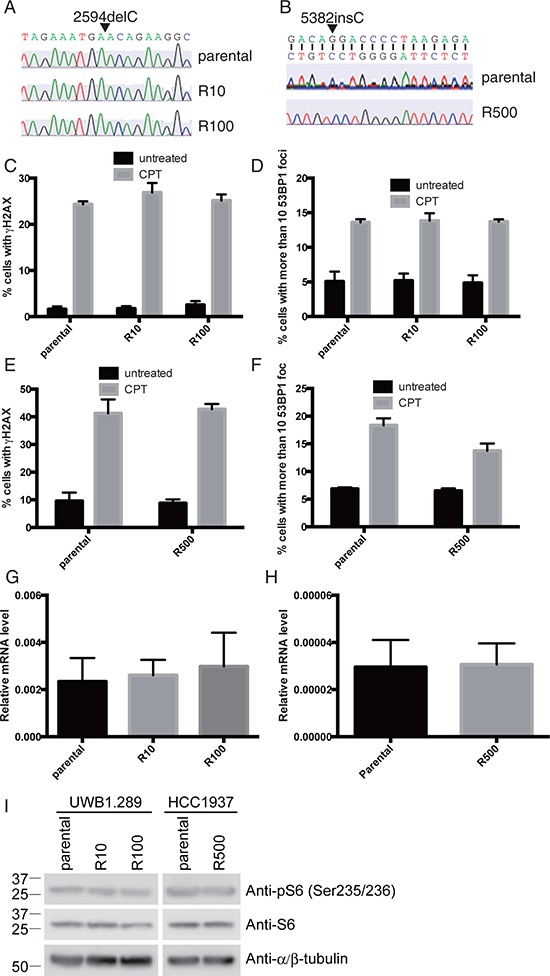
Known mechanisms for PARP inhibitor-resistance are not applicable Direct sequencing of *BRCA1* gene in UWB1.289 **(A)** and HCC1937 **(B)** are shown. Cells were treated with CPT (camptothecin: 3μM) for 3 hours and stained with antibodies against γH2AX and 53BP1. Untreated cells were also stained as control. Histogram shows ratio of γH2AX positive cells in UWB1.289 **(C)** and in HCC1937 **(E)** and ratio of cells with more than 10 53BP1 foci in UWB1.289 **(D)** and in HCC1937 **(F)**. Error bar shows standard deviation of three independent experiments. Histogram shows mRNA expression of *Abcb1a* in parental and PARP inhibitor-resistant UWB1.289 **(G)** or HCC1937 **(H)**. Error bar shows standard deviation of three independent experiments. Cell lysates from parental or PARP inhibitor-resistant UWB1.289 or HCC1937 cells were subjected for western blotting with indicated antibodies **(I)**.

Several mechanisms for acquired resistance to PARP inhibition have been already proposed [5–9]. Therefore, prior to exploring a new mechanism with a comprehensive screening in PARP inhibitor-resistant cells, we have tested whether any of these reported mechanisms were responsible for PARP inhibitor-resistance in our PARP inhibitor-resistant lines. One reported mechanism for acquired resistance to PARP inhibition is a reversion of *BRCA1* gene mutation [6]. Therefore we checked the sequence of *BRCA1* gene. The mutations of *BRCA1* genes are conserved in all the resistant lines (Figure 2A and 2B). Another reported mechanism is disruption of 53BP1 function [7]. Treatment with DNA damaging agents such as camptothecin, a topoisomerase I inhibitor, induces 53BP1 foci formation when 53BP1 is functional [17]. All the parental and PARP inhibitor-resistant UWB1.289 or HCC1937 cells display similar level of induction of 53BP1 foci formation with elevation of γH2AX signal, a surrogate marker of DNA double strand breaks, in response to camptothecin (Figure 2C–2F and Supplementary Figure 1) [18]. These results suggest that 53BP1 is functional in the PARP inhibitor-resistant lines. Other reports suggest that up-regulation of p-glycoprotein efflux pump *Abcb1a* (as defined by mRNA expression), is a possible mechanism for PARP inhibitor-resistance [8]. mRNA expression of *Abcb1a* in parental and PARP inhibitor-resistant UWB1.289 or HCC1937 cells was investigated and the expression of the *Abcb1a* is not altered through the lines (Figure 2G and 2H). An additional mechanism reported for resistance to PARP inhibition is excess phosphorylation of ribosomal protein S6 [9]. However, this is also not applicable in our PARP inhibitor-resistant clones (Figure 2I). Therefore we explored a new mechanism for resistance to PARP inhibition using our clones with acquired resistance, using a comprehensive screening approach.

### RNA sequencing to compare PARP inhibitor-resistant and sensitive clones of the ovarian cell line UWB1.289

We performed whole transcriptome RNA sequencing of cDNA libraries derived from parental UWB1.289, and the PARP inhibitor-resistant clones R10 and R100. We obtained reads to the order of ~13 million (parental) and ~14 million (R10 and R100) (Supplementary Figure 2). Among these reads of parental, R10 and R100, 81 to 85% of them were mapped to human genome GRCh37. Using Rsem and EdgeR analysis, we identified differentially expressed genes (DEGs) among the parental, R10 and R100 (parental vs R10 and parental vs R100) [19, 20].

### Pathway analysis for acquired resistance to PARP inhibition

Next we used Ingenuity Pathway Analysis (IPA) to identify the DEGs in the common pathways. IPA is a web-based software to analyze RNA sequencing data to understand relation to large biological systems [21]. In order to obtain meaningful output, we identified DEGs by log ratio ≥ 2 and *p*-value ≤ 5.00E-02. In this setting, we identified 118 DEGs (parental vs R10) and 85 DEGs (parental vs R100). IPA with DEGs for parental vs R10 or parental vs R100 shows significant enrichment of genes in category of “Cancer” among the other categories (Supplementary Table 1). To identify the exact pathway in which DEGs are involved, we performed a pathway analysis in IPA. Interestingly, 50% or above of DEGs involved in both the pathways are genes regulated by NF-κB signaling (4 genes out of 7 DEG in R10 vs parental and 4 genes out of 8 DEGs in R100 vs parental, highlighted in bold letter in Table 1). The DEGs were further validated by network analysis by IPA. This analysis reveals that the top differential networks are “Collagen type 1-” and “TNF family-” in parental vs R10 and parental vs R100, respectively (Table 2). Again both the networks are enriched in genes regulated by NF-κB signaling (highlighted in bold letter in Table 2). IPA predicts upstream transcriptional regulators from the global change of mRNA expression. The upstream prediction also reveals enrichment of NF-κB regulators such as TNF, STAT3, IL1 and IL1A (Table 3). In summary, IPA analysis predicts that up-regulation of NF-κB signaling occurs in PARP inhibitor-resistant cells, on the basis of up-regulation of genes regulated by NF-κB signaling. Aside from the upstream classification of DEGs, we have also tried to identify common downstream signaling pathways that DEGs are involved using the Kyoto Encyclopedia of Genes and Genomes (KEGG) database. While IPA performs analysis with DEGs that are both up-regulated and down-regulated simultaneously, KEGG performs analysis with DEGs either up- or down-regulated. Therefore we picked up up-regulated DEGs defined as log ratio ≥ 1.5 and *p*-value ≤ 5.00E-02 for analysis with KEGG. This threshold generates 307 and 314 DEGs in parental vs R10 and parental vs R100, respectively. This analysis revealed that pathways of cytokine-cytokine receptor interaction and cell adhesion molecules (CAMs) are significantly enriched (Table 4). In particular, cytokines and chemokines that are upstream effectors as well as downstream products of NF-κB signaling, such as IL1β and CCL20, are up-regulated in PARP inhibitor-resistant cells (both in R10 or R100) compared to the parental. These results suggest that NF-κB pathway is preferentially activated in PARP inhibitor-resistant cells.

**Table 1:** Top “Canonical Pathways” from the RNA-seq by IPA

Parental *vs*. R10			
Biological process	*p*-value	Ratio	Contributing genes in dataset
Hepatic Fibrosis / Hepatic Stellate Cell Activation	1.4E-04	7/197 (0.036)	**CCL2** (2.824), **COL1A2** (4.786), IGFBP5 (3.739), MYH14 (6.017), MYL2 (3.343), **NGFR** (2.696), **TNFSF18** (2.427)

Parental *vs*. R100			
Biological process	*p*-value	Ratio	Contributing genes in dataset
Agranulocyte Adhesion and Diapedesis	9.14E-07	8/189 (0.042)	**CCL2** (2.989), **CCL20** (3.293), CDH5 (2.123), IL36G (2.719), **MMP7** (2.409), MYH14 (4.215), MYL2 (2.390), **SELL** (2.407)

Genes involved directly in NF-κB including TNF signaling are shown in bold letter.

The *p*-value was calculated by Fisher's exact test.

The ratio shows the number of DEGs involved in the pathway divided by total number of genes making up that pathway.

**Table 2:** Top “Networks” from the RNA-seq by IPA

Parental *vs*. R10			
Molecules in network	Score	Focus Molecules	Top disease and functions
ADAMTS5, **Alp**, Alpha, catenin, Atrial Natriuretic Peptide, BSCL2, Cadherin, CDH4, CDH5, CDH11, Cg, CNN1, **Collagen type I**, Collagen(s), DGKI, **ERK1/2**, Fibrin, FMOD, GDF6, HSD3B7, IGFBP5, KRT17, Laminin, MSX1, NPR1, NPR3, PDGFBB, Secretaseγ, STEAP4, SULF1, **TGFβ**, THY1, TLL1, **TNFAIP6**, **VCAN,** Wnt	42	21	Cell Morphology, Carbohydrate Metabolism, Drug Metabolism

Parental *vs*. R100			
Molecules in network	Score	Focus Molecules	Top disease and functions
**Alp**, Alphacatenin, Cadherin, **CCL20**, CD3, CDH4, CDH11, CEACAM1, Collagen(s), DDC, DGKI, **ERK1/2**, Fibrin, FMOD, GDF6, **IFNγ**, IGFBP5, **IL1**, IL36G, Laminin, **LGALS9**, Mek, **Mmp**, POSTN, **SELL**, Sos, STEAP4, SULF1, **TGFβ**, **TNF(family)**, **TNF receptor**, **TNFAIP6**, **TNFSF18**, **TRAF1**, **VCAN**	40	19	Carbohydrate Metabolism, Drug Metabolism, Small Molecule Biochemistry

Genes involved directly in NF-κB including TNF signaling are shown in bold letter.

The score indicates reliability of the network that DEGs are involved (<20: less reliability and >40: high reliability). The score is calculated based on the hypergeometric distribution and is a negative log of the *p*-value (score = −log10 (*p*-value)).

**Table 3:** Top 5 “Upstream regulators” from the RNA-seq by IPA

Parental *vs*. R10			
Upstream Regulator	Activation z-score	*p*-value of overlap	Target molecules in dataset
Tretinoin	2.928	9.49E-04	ADAMTS5, ALDH1A2, **CCL2**, CDH5, **COL1A2**, CYP4B1, FAM153A/FAM153B, FOLR2, IGFBP5, KITLG, **LGALS9**, LY6E, MYL2, POSTN, PTF1A, RARRES2, **TNFAIP6**
**TNF**	2.814	3.12E-03	ADAMTS5, **CCL2**, CDH11, CNN1, **COL1A2**, DSC3, IGFBP5, KITLG, LGALS9, **NGFR**, NNMT, **P2RY6**, POSTN, RARRES2, STEAP4, THY1, **TNFAIP6**
IFNG	2.442	3.17E-02	BST1, **CCL2**, CECR1, **COL1A2**, KITLG, KRT17, **LGALS9**, LY6E, MX2, **P2RY6**, THY1, **TNFAIP6**
Decitabine	2.138	7.20E-04	ALDH1A2, CDH11, CDH4, **COL1A2**, CYP4B1, KRT75, NPTX1, TLL1, **VCAN**
**STAT3**	1.982	2.24E-03	**CCL2**, CDH5, **COL1A2**, FLRT3, IGFBP5, KRT17, MX2, **VCAN**

Parental *vs*. R100			
Upstream Regulator	Activation z-score	*p*-value of overlap	Target molecules in dataset
**TNF**	2.894	3.81E-05	**CCL2, CCL20**, CDH11, CNN1, **COL1A2**, DSC3, IGFBP5, IL36G, **LGALS9**, **MMP7**, **NGFR**, NNMT, **P2RY6**, POSTN, STEAP4, **TNFAIP6**, **TRAF1**
IFNG	2.645	1.89E-03	**CCL2**, **CCL20**, CEACAM1, **COL1A2**, IL36G, **LGALS**, LY6E, MX2, **P2RY6**, SELL, **TNFAIP6**, **TNFRSF14**
poly rI:rC-RNA	2.538	7.87E-04	**CCL2**, **CCL20**, **LGALS9**, LY6E, **TNFAIP6**, **TRAF1**, TRIM6-TRIM34
**IL1**	2.509	3.68E-07	**CCL2**, **CCL20**, CEACAM1, DDC, GCK, **MMP7**, **NGFR**, **SELL**, **TNFAIP6**, **VCAN**
**IL1A**	2.367	1.18E-04	**CCL2**, **CCL20**, IGFBP5, IL36G, **LGALS9**, **P2RY6**

Genes involved directly in NF-κB including TNF signaling are shown in bold letter.

Activation z-score indicates activation state of transcriptional regulators. This is based on expression of downstream genes. Above/below 1 means activation/inhibition of the transcriptional regulator.

Overlap *p*-value indicates significant overlap between DEGs and a transcriptional regulator that could regulate the DEGs.

**Table 4:** Top 5 “Upstream regulators” from the RNA-seq by KEGG

Parental *vs*. R10			
KEGG pathway	*p*-value	No. of DEG involved / No. of genes in pathway	Contributing genes in dataset
hsa04514:Cell adhesion molecules (CAMs)	0.0014	7/132	CDH5, CDH2, NCAM, NGL1, **SELL**, SDC, **VCAN**
hsa05410:Hypertrophic cardiomyopathy (HCM)	0.0039	5/85	DHPR, TnC, MYL2, ACE1, **TGFβ**
hsa04060:Cytokine-cytokine receptor interaction	0.043	9/262	**CCL20**, **CCL2**, **CX3CL1**, KITLG, **NGFR**, **SF14**, **TNFSF18**, **TGFB2**, IL1R2
hsa04610:Complement and coagulation cascades	0.086	4/69	Coagulation factor III (thromboplastin, tissue factor), complement component 1, subcomponent, proteins (aopha), serpin peptidase inhibitor clade E (nexin plasminogen activator inhibitor type 1) member1

Parental *vs*. R100			
KEGG pathway	*p*-value	No. of DEG involved / No. of genes in pathway	Contributing genes in dataset
hsa04060:Cytokine-cytokine receptor interaction	3.13E-06	19/262	**CXCL3**, **CXCL5**, **CXCL10**, **CXCL1**, **CCL2**, **CCL20**, IL23A, CSF2, KITLG, FLT1, **TNFSF15**, **TNFSF18**, **SFIIB**, **NGFR**, **SFIB**, **SF14**, **SF9**, **TGFB2**, INHBA
hsa04070:Phoshatidylinositol signaling system	0.016	6/74	Diacylglycerol kinase, iota, inositol 1,4,5-triphoshate3-kinaseA, phospholipase C, beta 1(phosphoinositide-specific), phospholipase C, delta 1, synaptojanin 2
hsa00562:Inositol phosphate matabolism	0.022	5/54	Inositol 1,4,5-triphoshate3-kinase A, Phospholipase C, beta 1 (phosphoinositide-specific), phospholipase C, delta 1, synaptojanin 2
hsa04512:ECM-receptor interaction	0.026	6/84	**Collagen type1 alpha2**, Reelin, THBS, Syndecan, CD47, Collagen Type VI alpha 1
hsa04510:Focal adhesion	0.048	9/201	Baculoviral IAP repeat-containing 3, **collagen type1 alpha2**, collagen type VI alpha1, fms-related tyrosine kinase, myosine light chain kinase, myosine light chain 2, Platelet derived growth factor D, reelin, thrombospondinl
hsa04062:Chemokine signaling pathway	0.080	8/187	**CCL2**, **CCL20**, **CXCL10**, **CXCL3**, **CXCL5**, **CX3CL1**, adenylate cyclase4, phospholipase C beta (phosphoinositide-specific)

### NF-κB signaling is up-regulated in resistant cells

**Figure 3:**
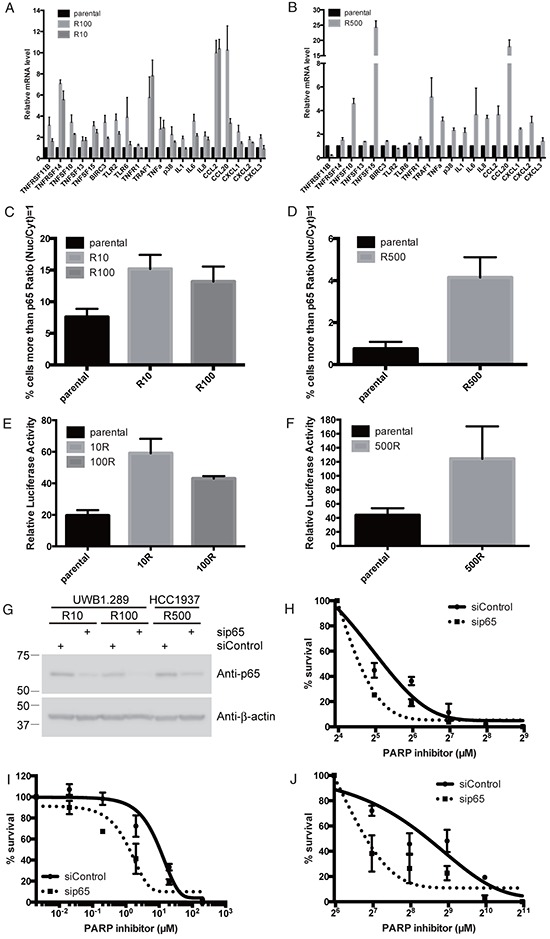
NF-κB is up-regulated in PARP inhibitor-resistant cells Histogram shows mRNA of indicated genes detected by RT-PCR in parental or PARP inhibitor-resistant UWB1.289 **(A)** or HCC1937 **(B)**. Error bar shows standard error of three independent experiments. Histogram shows percent of cells with p65 in nucleus in parental or PARP inhibitor-resistant UWB1.289 **(C)** or HCC1937 cells **(D)**. In C and D, p65 nuclear translocation was presented as % of cells with intensity contrast: (nuclear intensity – cytoplasmic intensity)/(nuclear intensity + cytoplasmic intensity) ≥ 1. Error bar shows standard deviation of three independent experiments. Histogram shows Luciferase activity of an NF-κB-luciferase reporter plasmid in parental or PARP inhibitor-resistant UWB1.289 **(E)** or HCC1937 **(F)** cells, presented relative to the activity of renilla luciferase. Y-axis indicates luciferase activity (relative). Error bar shows standard deviation of three independent experiments. PARP inhibitor-resistant UWB1.289 or HCC1937 cells were transfected with siRNA for p65 or non-targeting siRNA as indicated. Twenty-four hours post transfection, cells were reseeded into 6 well plates for western blotting as well as for clonogenic assay. Forty-eight hours post transfection, cell lysates were subjected for western blotting with indicated antibodies **(G)**, also cells were treated with different concentrations of PARP inhibitor (olaparib) as indicated. Line chart shows sensitivity to PARP inhibitor in transfected resistant R10 **(H)** and R100 **(I)** in UWB1.289 or R500 **(J)** in HCC1937 cells. Error bar shows standard deviation of three independent experiments.

To confirm that NF-κB signaling is up-regulated in PARP inhibitor-resistant cells, we performed quantitative Reverse Transcription-Polymerase Chain Reaction (RT-PCR) to measure upstream effectors and downstream transcription products of NF-κB signaling. From IPA and KEGG analysis, we selected a panel of genes involved in TNFα signaling and NF-κB signaling including TNF receptor family, TNF ligands superfamily members, anti-apoptotic genes, and genes that stimulate inflammatory response such as Toll-like receptor family, cytokines, chemokines and p38, an activator of NF-κB through STAT3 activation (see Supplementary Table 2). Almost all these genes are indeed increased in both the PARP inhibitor-resistant cells (R10 and R100) compared to parental UWB1.289 cells (Figure 3A). RT-PCR shows almost the same agreement with RNA sequencing data, which is 95% similarity (19 of 20 genes). We also attempted to validate these findings using the breast HCC1937 cell lines (parental and R500). Interestingly, while the mRNA of TNF ligands superfamily members, p38, cytokines and chemokines are increased in R500 compared to parental cells, TNF-receptor family members, anti-apoptotic genes or genes that stimulate inflammatory response are not altered in the resistant cells compared to parental (Figure 3B). However 70% of the list of the genes involved in NF-κB signaling increases the expression in PARP inhibitor-resistant cells. These results suggest that NF-κB signaling is up-regulated in PARP inhibitor resistant cells.

In canonical NF-κB signaling, to activate the transcription of target genes, the p65/p50 subunits of NF-κB are translocated into the nucleus [10]. Therefore we measured nuclear retention of p65 in parental or resistant cells, using quantitative high content microscopy. Prior to the experiment, we checked the quality of the anti-p65 antibody and the quantitative high content microscopy. For the quality check of the antibody, p65 was knocked down using siRNA in UWB1.289 cells and the cells were stained with the anti-p65 antibody. The signal of p65 is significantly reduced by the siRNA for p65 (Supplementary Figure 3A). We proceeded to measure the nuclear-cytoplasmic ratio of p65 using NucTrans.V4 algorithm in the cellomics HCS system. For a quality check of our quantitative high content microscopy protocols, the effect of a stimulator and an inhibitor of NF-κB signaling were measured. TNFα is used as a stimulator and BAY 11–7082, an IKKα inhibitor is used as an inhibitor of the signal [22]. Indeed, TNFα increases nuclear retention of p65, as defined by our protocol, and the effect of TNFα is inhibited by treatment with BAY 11–7082 (Supplementary Figure 3B and 3C). Therefore, we have performed further experiments using these settings. In UWB1.289 cells, ~7.5 percent of cells have nuclear accumulation of p65 in parental cells. In contrast, ~15.2 or ~13.2 percent of cells have accumulated p65 in nucleus in resistant R10 or R100 cells (Figure 3C and Supplementary Figure 4A). Similar results are observed in HCC1937. There is more p65 in nucleus in resistant R500 cells than in parental cells (~4.1 percent *vs*. ~0.6 percent, respectively) (Figure 3D and Supplementary Figure 4B). Furthermore, we measured NF-κB activation with NF-κB responsive luciferase reporter gene assay. TNFα induces luciferase activity and the effect of the TNFα is diminished by treatment with BAY 11–7082 (Supplementary Figure 3D), confirming the validity of this assay. In UWB1.289 cells, PARP inhibitor-resistant cells show a more than two fold increase in transcriptional activity (~19.5 in parental to ~59.1 or ~43 in R10 or R100 respectively) (Figure 3E). In HCC1937 cells, R500 shows almost 3 fold increase compared to parental cells (~43.7 in parental to ~124.3 in R500) (Figure 3F). Thus these results suggest that at least a subset of NF-κB signaling is indeed up-regulated in PARP inhibitor-resistant cells. Finally, we confirm the effect role of NF-κB signaling in mediating PARP inhibition by knockdown of p65, a central component in NF-κB signaling in sensitivity to PARP inhibitor. Strikingly, reduction of p65 reverses the sensitivity to PARP inhibitor in PARP inhibitor-resistant lines both in UWB1.289 and HCC1937 (Figure 3G–3J).

### PARP resistant cells are sensitive to NF-κB inhibition, and Bortezomib treatment

**Figure 4:**
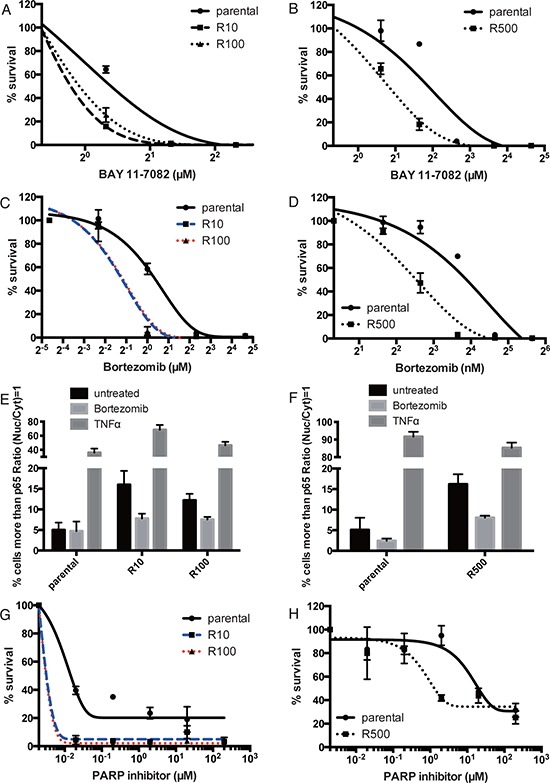
PARP inhibitor-resistant cells are sensitive to NF-κB inhibition Line chart shows sensitivity to BAY 11–7082 in parental or PARP inhibitor-resistant UWB1.289 **(A)** or HCC1937 cells **(B)**. Error bar shows standard deviation of three independent experiments. Line chart shows sensitivity to bortezomib in parental or PARP inhibitor-resistant UWB1.289 **(C)** or HCC1937 cells **(D)**. Error bar shows standard deviation of three independent experiments. The parental and PARP inhibitor-resistant UWB1.289 or HCC1937 cells were treated with bortezomib (500pM) for 24 hours or TNFα (100ng/ml) for 20 minutes and stained with anti-p65 antibody. Untreated cells were also stained as control. Histogram shows percent of cells with p65 in nucleus in parental or PARP inhibitor-resistant UWB1.289 **(E)** or HCC1937 **(F)** cells. p65 nuclear translocation was presented as % of cells with intensity contrast: (nuclear intensity – cytoplasmic intensity)/(nuclear intensity + cytoplasmic intensity) ≥ 1. Error bar shows standard deviation of three independent experiments. Line chart shows the sensitivity to PARP inhibitor (olaparib) with bortezomib (30nM for UWB1.289 and 500pM for HCC1937) in parental or resistant UWB1.289 **(G)** or HCC1937 **(H)** Error bar shows standard deviation of three independent experiments.

In accordance with a central role for NF-κB signaling in mediating resistance to PARP inhibitors, we show that the PARP inhibitor-resistant UWB1.289 or HCC1937 clones (R10, R100 and R500) are sensitive to an NF-κB inhibitor BAY 11–7082 compared to the parental cells (Figure 4A and 4B). Bortezomib a proteasome inhibitor, also impacts on the NF-κB pathway through protection of IκBα from the proteolysis by ubiquitin proteasome system [23]. Whether bortezomib works as an inhibitor of NF-κB signaling is controversial [24, 25], but it has been demonstrated that bortezomib kills cells with up-regulated NF-κB signaling [26]. As the drug is currently approved by the U.S. Food and Drug Administration for clinical use in the treatment of Multiple Myeloma [27], we checked the cellular sensitivity to bortezomib in UWB1.289 or HCC1937 cells. As expected, bortezomib kills PARP inhibitor-resistant cells derived from UWB1.289 preferentially compared to the parental cells (Figure 4C). This effect is also observed in HCC1937 (Figure 4D). We further tested whether bortezomib inhibits or activates NF-κB signaling in the PARP inhibitor-resistant cells. Bortezomib indeed decreases nuclear retention of p65 preferentially in PARP inhibitor-resistant lines compared to parental UWB1.289 (Figure 4E) or HCC1937 cells (Figure 4F), suggesting that it seems to inhibit NF-κB signaling in the setting of acquired PARP resistance.

This prompted us to check if bortezomib could be used to reverse the PARP resistant phenotype of our cell lines. The sensitivity to bortezomib in parental and PARP inhibitor-resistant clones does not differ up to 200nM in UWB1.289 and 4nM in HCC1937 (Figure 4A and 4B). Therefore the sensitivity to PARP inhibition in conjunction with a low dose of bortezomib (30nM for UWB1.289 and 500pM for HCC1937) was assessed. As expected, bortezomib increases the sensitivity to PARP inhibition in PARP inhibitor-resistant cells preferentially (Figure 4G and 4H). These data suggest that inhibition of NF-κB signaling is an effective therapy for PARP inhibitor-resistant cancers, and that inhibition of NF-κB signaling reverses cellular sensitivity to PARP inhibition in resistant cells.

## DISCUSSION

The mechanism of acquired resistance to PARP inhibitor has been extensively studied and several models have been proposed [5–9]. Because all the mechanisms suggested were not applicable in our resistant lines, we screened an ovarian cancer cell line (which is the most clinically applicable context for continuous PARP inhibitor therapy [4]) by RNA sequencing, and note increased NF-κB pathway activation. Interestingly, up-regulation of NF-κB signaling is known to cause acquired resistance to other agents apart from PARP inhibitors [13].

We have shown that mRNA of genes that are involved in NF-κB signaling are altered in expression when the cells acquire resistance to PARP inhibition. The precise mechanism by which these NF-κB related mRNAs are up-regulated is not known, and will be the subject of future work in the lab.

NF-κB has an anti-apoptotic effect and it has been thought that increased anti-apoptotic effect by up-regulated NF-κB signaling plays an important role in acquired resistance [13]. Interestingly, genes that are involved in anti-apoptosis are not up-regulated both in UWB1.289 and HCC1937 in our setting. How up-regulated NF-κB signaling without up-regulation of anti-apoptotic gene serves for the acquired resistance is not known. The information of the exact mechanism may help to develop a better therapy for chemoresistant malignancies.

Finally, we propose inhibition of NF-κB is an effective anti-cancer therapy for malignancies for acquired resistance to PARP inhibition. NF-κB inhibitors are still not used as practical clinical medicines because of toxicity and specificity [28]. On the other hand, while bortezomib is not a typical NF-κB inhibitor, it does have a negative influence on the pathway in most contexts, and is clinically approved [27]. Furthermore, co-treatment with bortezomib may allow a reversal of the acquired resistance to PARP inhibition. Further study is needed to develop this idea for clinical use to benefit patients who progress on PARP inhibitor therapy.

## MATERIALS AND METHODS

### Cell culture

UWB1.289 and HCC1937 cells were cultured in RPMI-1640 medium supplemented with 10% fetal bovine serum and 1% penicillin-streptomycin at 37°C.

### Generation of PARP inhibitor-resistant clones

To generate PARP inhibitor-resistant clones, UWB1.289 cells were continuously exposed to different doses of PARP inhibitor (10nM or 100nM). The cells were split every other day for 5 months. In HCC1937, cells were exposed to PARP inhibitor (500nM). The cells were split every other day for 3 months.

### Cell viability assay

Cells were plated into 6-well plates at a density of 1000 cells per well. Different doses of drugs were added, and the plates were incubated at 37°C for a week. Cells were fixed with 75% methanol in 25% acetic acid for 5 min, and the plates were dried. Colonies were stained with Lillie's crystal violet (2 g crystal violet, 0.8 g ammonium oxalate in 100 ml of 80% ethanol) for 5 min and subsequently washed with water, dried, and measured by ImageQuant LAS 4000 (GE healthcare).

### Genomic DNA collection

Cells were lysed with 0.5% NF-40 lysis buffer. Once cells were dissolved, same volume of 100% phenol TE was added, then centrifuged at maximum speed. Supernatant was collected and same volume of isopropanol was added, and mixed well, then centrifugation. The pellet was washed with 70% ethanol once and dissolved with appropriate volume of water.

### Direct sequencing of *BRCA1* gene

Direct sequencing was performed by Applied Biosystems 3130 Genetic Analyzer following the protocol as suggested by the manufacturer. Primers for sequencing are CTGGTACTGATTATGGCACTCAGG for UWB1.289 and CTTAAAGTCCCAGCTCTTCCAC for HCC1937.

### Immunofluorescence

Cells were cultured on 96 well plastic plates (BD Falcon). 24 hours later, cells were treated with 3uM of Camptothecin for 1 hour, when required. The cells were fixed with 4% PFA in PBS for 15 minutes. Then cells were washed with PBS and permeabilized with 0.2% Triton-X100 for 5 minutes and blocked with 3% BSA in PBSt for 15 minutes. Followed by blocking, cells were incubates with primary antibodies for 1 hour at room temperature, then cells were washed three times with PBSt followed by incubated with secondary antibodies for 30 minutes at room temperature. Nuclei were stained with Hoechest 33342 (1:1000, Invitrogen) for 15 minutes and analyzed by Cellomics Cellinsight high content screening reader (Thermo Scientific).

### Antibodies

The antibodies and dilution used in this study were: Anti-γH2AX (Ser139) antibody (Millipore, 05–636, 1:1000); Anti-53BP1 antibody (Novus Biologicals, NB100–304, 1:1000); Anti-ribosomal protein S6 antibody (Cell Signaling Technology, 2217, 1:1000); Anti-phospho-ribosomal protein S6 (Ser235/236) antibody (Cell Signaling Technology, 4858, 1:2000); and Anti-α/β-tubulin antibody (NeoMarkers, DM1A, 1:5000); Anti-p65 antibody (Cell Signaling Technology, 8242, 1:500 for IF and 1:1000 for WB); Anti-β-actin (SIGMA, AC-15, 1:1000); secondary antibodies (Alexa Flour, 1:1000).

### Real-time reverse transcription polymerase chain reaction

Quantitative real-time RT-PCR of transcript levels in UWB1.289 and HCC1937 were performed using a StepOnePlus™ real time PCR system (applied biosystems, Warrington, UK). Total RNA was extracted using an RNeasy Mini Kit (QIAGEN Sciences, Valencia, CA), and cDNA was synthesized using a PrimeScriptTM RT Master Mix (Takara, Tokyo, Japan) according to the manufacturer's protocol. Real-time PCR was performed using Power SYBR Green PCR Master Mix (applied biosystems) as follows: 15 min at 95C and then 45 cycles of 15 sec at 95C and 60 sec at 60C. Data were analyzed by the cycle threshold method to determine the fold changes in expression. Relative abundance of specific genes was normalized to those of GAPDH levels. Primers sequences used in Figure 3 are presented in Supplementary Table 2. Primers for *Abcb1a* gene are GAACAAGGGGAGCACCAAC (forward primer) and TGCTTTCCTCAAAGAGTTTCTG (reverse primer).

### Western blots

UWB1.289 and HCC1937 cells were transfected with siRNA using Lipofectamin RNAiMAX (Life Technologies) transfection reagent following manufacturer protocol. RNAi for p65 and non-targeting siRNA were purchased from Cell Signaling Technology. Western blots were done as described previously [29], briefly 48 hours after transfection, cells were lysed with 0.5% NP-40 lysis buffer (50mM Tris-HCl pH7.5, 150mM NaCl, 0.5% NP-40, 50mM NaF, 1mM DTT, 1mM Na_3_VO_4_, complete protease inhibitor cocktail (Roche) and 1mM PMSF) and resolved by SDS-PAGE

### RNA quality control and library preparation

Total RNA was quantified and purity checked using a NanoDrop ND-1000 (Thermo Scientific, Waltham, MA, USA). RNA integrity was verified using an Agilent 2100 Bioanalyzer (Agilent Technologies, Santa Clara, CA, USA). Subsequently, 500 ng of total RNA were used with the TruSeq RNA Sample Preparation v2 Kit (Illumina, San Diego, CA) to construct index-tagged cDNA libraries. Libraries were quantified using a Quant-iT™ dsDNA HS assay with the Q-bit fluorometer (Life Technologies, Carlsbad, California). Average library size and the size distribution were determined using a DNA 1000 assay in an Agilent 2100 Bioanalyzer. Libraries were normalized to 10 nmol/L using Tris-Cl 10 mmol/L, pH8.5 with 0.1% Tween 20.

### RNA sequencing and data analysis

Total RNA derived from PARP inhibitor-resistant or parental UWB1.289 cells were isolated by trizol and whole transcriptome analysis was performed. The sample of each cells were technical replicated. Cutadapt was used to trim Illumina adapters and to remove those reads that were too short. Filtered reads were then aligned with Rsem against the GRCh37.p11 collection of transcripts. Each RefSeq gene's expression was summarized and normalized using EdgeR bioconductor R package. Differentially expressed genes were identified with coverage (more than 1 at least one sample) and fold change (more than 1.5). Biological functions and network analysis of differentially expressed genes were performed using KEGG that is included in DAVID gene ontology (http://david.abcc.ncifcrf.gov/) as well as Ingenuity Pathway Analysis (IPA) (http://www.ingenuity.com/) [30, 31].

### Ingenuity Pathway Analysis (IPA)

RNA sequencing data were analyze by IPA software in terms of search common networks and canonical pathways. Significance of the networks and canonical pathways were tested by the *p*-value. Top networks show associative networks based on a score. The statistically significance were considered by score ≥ 2.

### Plasmids

For NF-κB activation assays in UWB1.289 and HCC1937 cells, we used an NF-κB site–containing luciferase reporter plasmid. For normalization and control, we used a luciferase reporter without the NF-κB site–containing and a *Renilla* luciferase for normalization. All the constructs are under pRL-TK backbone vectors (A gift from Dr. Grahame McKenzie).

### Luciferase assay

5 × 10^6^ of UWB1.289 cells or 3 × 10^6^ of HCC1937 cells were transfected with 14ug of NF-κB firefly luciferase reporter vector and 1μg of pRL-TK renilla luciferase vector. For control, UWB1.289 or HCC1937 cells were transfected with 14μg of pGL3 basic firely luciferase reporter vector with 1μg of pRL-TK renilla luciferase vector. Transfections were carried out with Cell Line Nucleofector Kit V in Nucleofector (Lonza) using program A-023. Cells were cultured in 2ml of RPMI-1640 medium in a 12-well plate. Luciferase assay was performed using Dual Luciferase Assay kit (Promega) per manufactures instructions. Luciferase activity was measured at 48 hours post transfection. For measurement, 30μl of each renilla and firefly substrate were injected into 30μl out of 120μl of cell lysate. Luciferase signals of firefly were divided by renilla signals and standard error of the mean (SEM) of triplicated experiments was calculated.

### Statistical analysis

All the statistical analyses have been done by graphpad prism. The statistically significance were considered by *p* < 0.05.

## SUPPLEMENTARY FIGURES AND TABLES


