# Supplementary material for: NF-κB signaling mediates acquired resistance after PARP inhibition
Source: Oncotarget. 2015 Jan 13;6(6):3825–39. doi: 10.18632/oncotarget.2868 (PMC4414156; doi:10.18632/oncotarget.2868)
Supplement: Supplementary file 1 [file oncotarget-06-3825-s001.pdf]

SUPPLEMENTARY FIGURES AND TABLES

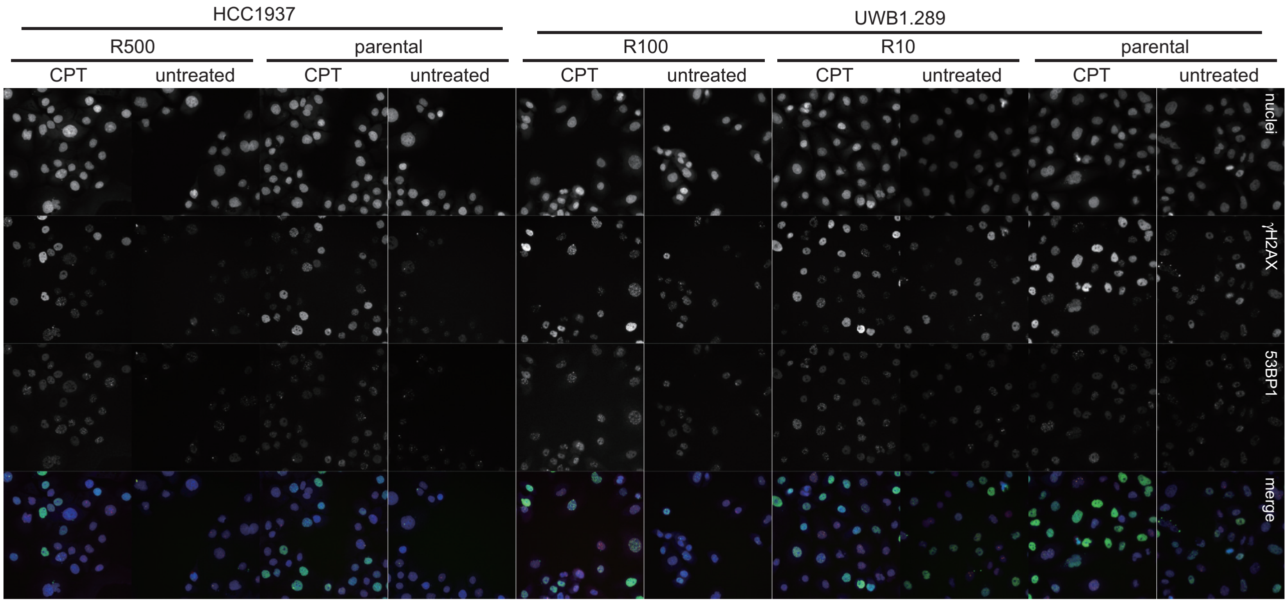

Supplementary Figure 1: 53BP1 foci formation is induced in resistant cells. Both parental and resistant UWB1.289 or HCC1937 cells were treated with CPT (Camptothecin: 3μM) for 1 hour and stained with antibodies against γH2AX and 53BP1. Untreated cells were also stained as control. A representative images is shown.

Raw Illumina Reads

Sample	Raw reads
parental_1	11725551
parental_2	13711733
R10_1	14231430
R10_2	13779221
R100_1	14744042
R100_2	11183701

↓

Filtered Reads

Sequencing quarity check by FastQC
Triming of Illumina adapters by Cutadapt

Sample	Pre-processed reads
parental_1	11406138
parental_2	13542692
R10_1	13822231
R10_2	13341398
R100_1	14089236
R100_2	10952922

↓

Reads Mapping

Maping of the filtered reads by Rsem

Sample	Mapped Reads	% mapped reads
parental_1	9755224	83
parental_2	11627004	84
R10_1	11907508	83
R10_2	11475818	83
R100_1	11988152	81
R100_2	9373174	83

↓

Differentially Expressed Genes

Normalization of counts and
calculation of significance by Edge R

R10 vs parental
R100 vs parental

↓

Pathway Analysis
by IPA and KEGG

Supplementary Figure 2: A schematic flow chart of analysis of RNA sequencing. Rsem aligns RNA sequencing reads to genome reference (CRCh37.p11 collections of transcripts). Mapping counts calculated with Rsem were used to identify differentially expressed genes (DEGs) with Edge R. Two comparisons were performed (R10 *vs.* parental and R100 *vs.* parental), to analyze functional pathways by IPA as well as by KEGG analysis.

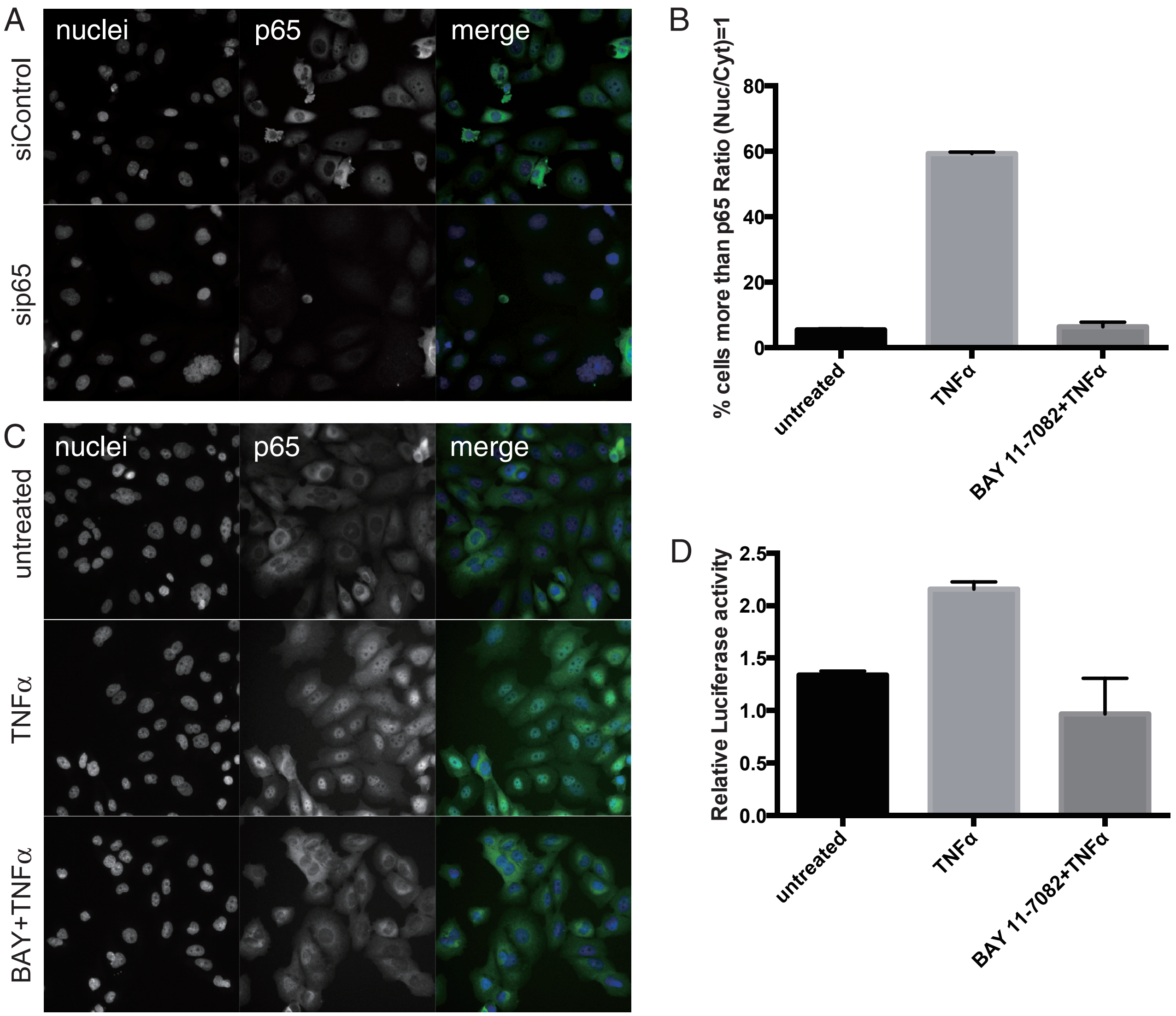

Supplementary Figure 3: Validation of the assay. **(A)** UWB1.289 cells were transfected with siRNA for p65. Forty-eight hours post transfection, cells were stained with anti-p65 antibody. Representative images are shown. **(B–C)** UWB1.289 cells were treated with TNFα (100ng/ml) for 20 minutes or BAY 11-7082 (10µM) for 1 hour followed by TNFα (100ng/ml) for 20 minutes. Cells were stained with anti-p65 antibody. Untreated cells were also stained as control. Histogram shows p65 nuclear translocation presented as % of cells with intensity contrast **(B)**. Error bar shows standard deviation of three independent experiments. Representative images are shown in **(C)** **(D)**. UWB1.289 cells were electroporated with and pRL-TK renilla luciferase vector in combination of pGL3-NF-κB firefly luciferase reporter vector or pGL3-basic vector as control. Twenty-four hours post transfection, the cells were treated with TNFα (20ng/ml) for 24 hours or BAY 11-7082 (1µM) for 12 hour followed by TNFα (20ng/ml) for 24 hours. Histogram shows Luciferase activity of an NF-κB–luciferase reporter plasmid in resistant or parental UWB1.289 cells, presented relative to the activity of renilla luciferase. Y axis indicates luciferase activity (relative). Error bar shows standard error of three independent experiments.

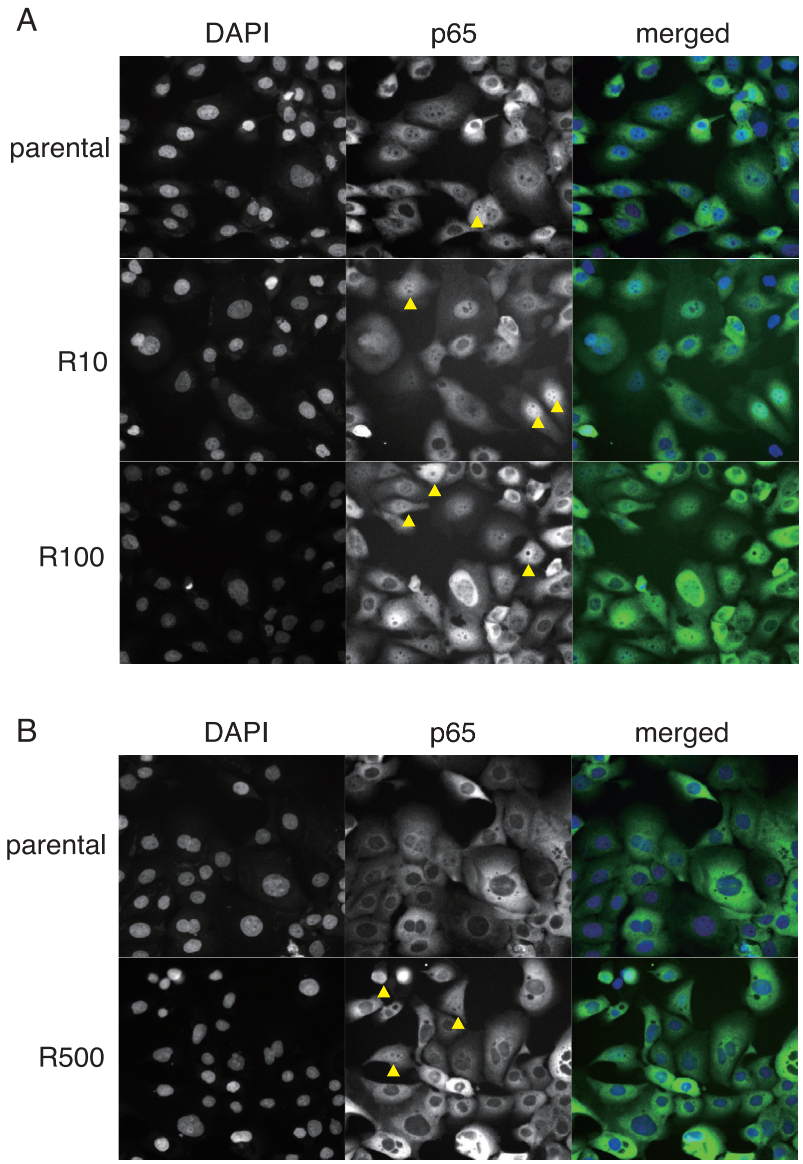

Supplementary Figure 4: Nuclear localization of p65 is increased in PARP inhibitor-resistant cells. Representative images of p65 in parental or PARP inhibitor-resistant (R10 and R100) UWB1.289 cells **(A)**, and in parental or PARP inhibitor-resistant (R500) HCC1937 cells **(B)** are shown. Yellow triangle indicates typical example of p65 in nucleus.

Supplementary Table 1: Top "Disease and Cellular functions" from the RNA-seq by IPA

**Parental *vs.* R10**		
**Diseases and Disorders**		
**Name**	***p*-value**	**#Molecules**
Cancer	2.39E-05 – 1.64E-02	75
Organismal Injury and Abnormalities	5.17E-05 – 1.64E-02	38
Reproductive System Disease	5.17E-05 – 1.64E-02	26
Cardiovascular Disease	1.09E-04 – 1.64E-02	29
Neurological Disease	2.54E-04 – 1.64E-02	32
**Molecular and Cellular Functions**		
**Name**	***p*-value**	**#Molecules**
Cell Morphology	3.00E-05 – 1.64E-02	25
Cell-To-Cell Signaling and Interaction	2.37E-04 – 1.64E-02	29
Small Molecule Biochemistry	2.37E-04 – 1.64E-02	29
Cellular Movement	4.77E-04 – 1.64E-02	31
Carbohydrate Metabolism	8.21E-04 – 1.64E-02	8

**Parental *vs.* R100**		
**Diseases and Disorders**		
**Name**	***p*-value**	**#Molecules**
Cancer	6.15E-06 – 1.55E-02	59
Cardiovascular Disease	1.49E-05 – 1.34E-02	19
Inflammatory Response	4.50E-05 – 1.45E-02	15
Organismal Injury and Abnormalities	1.79E-04 – 1.46E-02	30
Reproductive System Disease	1.79E-04 – 1.46E-02	23
**Molecular and Cellular Functions**		
**Name**	***p*-value**	**#Molecules**
Cellular Movement	9.06E-06 – 1.45E-02	26
Cellular Growth and Proliferation	3.96E-05 – 1.17E-02	36
Cell-To-Cell Signaling and Interaction<br>Small Molecule Biochemistry	2.23E-04 – 1.55E-02	31
Small Molecule Biochemistry	2.37E-04 – 1.52E-02	18
Carbohydrate Metabolism	4.15E-04 – 1.39E-02	11

Supplementary Table 2: Primer pairs for RT-PCR analysis

Function	Gene	Primers from 5′ to 3′
TNF receptor family member	TNFRSF11B	F:ACGGAGTTGCCACTTGACTTG<br>R:CCGGAAACAGTGAATCAACTC
	TNFRSF14	F:TTTGCTCCACAGTTGGCCTAATC<br>R:CAATGACTGTGGCCTCACCTTC
TNF ligand family member	TNFα	F:TCTTCTCGAACCCCGAGTGA<br>R:GGAGCTGCCCCTCAGCTT
	TNFSF10	F:TGCCAGGCAAATTGTCTACC<br>R:CGTGTACTTTACCAACGAGCTGA
	TNFSF13	F:ACTCTCAGTTGCCCTCTGGTTG<br>R:GGAACTCTGCTCCGGGAGACTC
	TNFSF15	F:GGACAGGAGTTTGCACCTTC<br>R:CTGTCAGGTGTGCCCTTGG
Anti-apoptotic	BIRC3	F:TGTTGGGAATCTGGAGATGA<br>R:CGGATGAACTCCTGTCCTTT
inflammatory response	TLR2	F:TGCAAGTATGAACTGGACTTCT<br>R:CCAGGTAGGTCTTGGTGTTCATT
	TLR6	F:GCAAAAACCCTTCACCTTGTTTTTC<br>R:CCAAGTCGTTTCTATGTGGTTGAGG
TNF receptor family member	TNFR1	F:ACTGCCTCAGCTGCTCCAAAT<br>R:CCGGTCCACTGTGCAAGAA
	TRAF1	F:GAGAACCCGAGGAATGGC<br>R:CTTCCCTTGAAGGAGCAGC
STAT3 activation	p38	F:CCAAACAGTGGATATCTGGTCC<br>R:CGGGCGTGTTCTGAGGAG
cytokine	IL1β	F:GCCCTAAACAGATGAAGTGC<br>R:GCCACAACAACTGACGCG
	IL6	F:GCCCTGAGAAAGGAGACA<br>R:CACCAGGCAAGTCTCCTC
chemokine	IL8	F:GTGTGAAGGTGCAGTTTTGC<br>R:CCATCAGAAAGCTTTACAATAATTTCT
CC chemokine	CCL2	F:GCTCGCTCAGCCAGATGC<br>R:CAATGGTCTTGAAGATCACAGC
	CCL20	F:CCACCTCTGCGGCGAATC<br>R:GTGAAAGATGATAGCATTGATGTCAC
CXC chemokine	CXCL1	F:GCGCGCAGCAGGAGCGT<br>R:GTTGGATTTGTCACTGTTCAGC
	CXCL2	F:CCCAAACCGAAGTCATAGC<br>R:GGATTTGCCATTTTTCAGC
	CXCL3	F:GAACAAGGGGAGCACCAAC<br>R:CTTACATTCACACTTTGGATGTTC
control	GAPDH	F:ACCCACTCCTACCTTTGA<br>R:CATACCAGGAAATGAGCTTGACAA
